# Electrochemotherapy in the treatment of locally advanced or recurrent eyelid-periocular basal cell carcinomas

**DOI:** 10.1038/s41598-019-41026-2

**Published:** 2019-03-12

**Authors:** Erika Gabriella Kis, Eszter Baltás, Henriette Ócsai, Attila Vass, István Balázs Németh, Erika Varga, Judit Oláh, Lajos Kemény, Edit Tóth-Molnár

**Affiliations:** 1 Department of Dermatology and Allergology, University of Szeged, Szeged, Hungary; 2 Department of Ophthalmology, University of Szeged, Szeged, Hungary; 3 MTA-SZTE Dermatological Research Group, Szeged, Hungary

## Abstract

There is increasing evidence about the effectiveness of electrochemotherapy (ECT) in the treatment of basal cell carcinomas in the head and neck region, although its role in the management of eyelid-periocular skin tumors has to be clarified. The aim of the present study is to evaluate the results of ECT in the treatment of locally advanced primary and recurrent eyelid-periocular skin basal cell carcinomas. Twelve patients with basal cell carcinoma involving the eyelid-periocular skin region were treated with ECT. Three patients had locally advanced primary tumors, while 9 patients had recurrent tumors. All treatments were performed according to the ESOPE guidelines, using Cliniporator TM device. All patients received bleomycin based ECT. The route of administration was intratumoral in 3 patients and intravenous in 9 patients. Tumor response was evaluated using the RECIST 1.1. criteria. ECT resulted in complete response of the periocular skin tumors in all patients. Lower eyelid ectropion was developed in 3 patients which had to be corrected surgically. ECT can be used effectively in the treatment of locally advanced or recurrent basal cell carcinomas in the eyelid-periocular skin region. Excellent tumor control can be achieved with good functional and cosmetic results without systemic adverse events with short interval follow-up.

## Introduction

Basal cell carcinoma (BCC) is the most frequently diagnosed skin malignancy in the eyelid-periocular skin region, representing approximately 90% of malignant skin tumors in this localization^1,2^. Treatment of locally advanced or recurrent BCC can be challenging^3^. Multiple factors must be taken into consideration when the most appropriate treatment modalities are planned. Complete elimination of the tumor must be achieved with acceptable functional and cosmetic results^4^. Inadequate treatment of advanced or recurrent periocular BCC can possess the risk of orbital invasion and therefore can jeopardize the eye or can be life threatening in case of intracranial propagation^5^. Our treatment options to achieve complete tumor control and to minimize the chance of recurrences have limitations. Surgical removal of large tumors can result in extensive skin defects with difficulties in reconstruction and with the potential risk of long-lasting wound healing mostly in the elderly population with considerable comorbidities. Although irradiation has a high success rate with curative intent, recurrent tumors observed in previously irradiated areas represent another group of challenging cases^6,7^. Hedgehog signaling pathway inhibitor vismodegib is a novel and effective therapeutic option in locally advanced and metastatic BCC, but systemic adverse events can limit its long-lasting administration^8,9^.

Electrochemotherapy (ECT) has recently been successfully added to the existing treatments for skin and superficial soft tissue metastases and irresecable primary cutaneous tumors in the clinical practice^10–13^. This modality proved to be an efficient, safe and cost-effective therapeutic option. ECT uses electroporation to enhance the permeability of the cell membrane on a reversible manner. Short-term electric pulses with high intensity result in transient pore formation in the cell membrane which enables the delivery of large hydrophilic molecules to the cytosol^14^. Various drugs have been tested in terms of potentiation of their cytotoxic effects by electroporation. Bleomycin and cisplatin have been found to be the most effective compounds and therefore they are the most frequently used chemotherapeutic agents during ECT^15,16^. Although there are increasing evidences about the effectiveness of ECT in the head and neck region, only few patients with BCC of the eyelid treated with ECT have been reported so far^17^. In the present study we report our results of eyelid-periocular BCC cases treated with ECT.

## Patients and Methods

### Patients

Patients with locally advanced or recurrent eyelid-periocular BCC treated with ECT at the Department of Dermatology and Allergology, University of Szeged between May 2014 and November 2017 were included in the present study. The study was approved by the Institutional Review Board of the University of Szeged, and was conducted in accordance with the principles of the Declaration of Helsinki. All patients gave their written informed consent prior to treatments. Informed consents were obtained from all patients to publish identifying information/images in an online open access publication.

### Methods

Detailed dermatological and ophthalmological examinations were performed in case of all patients. Clinical characteristics of the tumors, including size, number, localization and type (primary or recurrent) of the lesions were recorded. All patients underwent incisional biopsy prior to ECT treatment. All treatments were performed according to the ESOPE guidelines, using Cliniporator TM (IGEA Ltd, Modena, Italy) device^18^. Every patient received bleomycin based ECT, the route of administration was intratumoral or intravenous. Electric pulses were applied by means of standard needle electrodes after 1 or 8 min following intratumoral or systemic bleomycin administration, respectively. Row or hexagonal needle electrodes were applied. The electrical parameters of treatments with row needle electrodes were 8 square wave pulses 1000 V/cm for 100 ms at 5 kHz, with hexagonal electrodes 4 square wave pulses 910 V/cm for 100 ms at 5 kHz. For adequate tumor electroporation, current was checked at each delivered electrical pulses for being sufficient (hexagonal array >1.5 Amper [A], linear array >1.0A). Depending on the number, location and size of the lesions, general anesthesia with endotracheal intubation or laryngeal mask were used. The treatment time was usually less than 30 minutes. After ECT treatment, all patients were observed in the hospital for one day. Nausea and flue like symptoms as potential systemic adverse events were monitored. Simple non-adhesive dressings were applied to the wounds, and antibiotic eye drops (ofloxacine) were prescribed for 6–10 days.

Tumor response was evaluated according to the Response Evaluation Criteria in Solid Tumors (RECIST) 1.1 criteria^19^. Complete response (CR) was diagnosed in case of disappearance of the target lesion, while partial response (PR) was defined when at least 30% decrease in the baseline sum of the longest diameter of the target lesion was observed. The criteria of ECT re-treatment was PR noticed 6 weeks after ECT treatment, and the time interval between ECT sessions were 2 months.

In case of 11 patients, biopsies were taken from the treated lesions for histological evaluation of tumor response (1 patient refused the sampling).

Patients were monitored after the treatments: they were examined and photo-documented twice in the first month, monthly in the following 5 months and bimonthly thereafter.

### Statistical analysis

SPSS software version 17.0 (SPSS, Chicago, IL, USA) was used for statistical analysis.

## Results

Between May 2014 and November 2017, 12 patients with eyelid-periocular BCCs were treated with ECT. All patients were Caucasian (7 male and 5 female) (mean patient age: 61.6 years, range: 11–86 years). The follow-up interval was between 15 and 56 months (median: 19 months). Summary of the treated cases can be found in Table 1.

**Table 1:** Summary of the eyelid-periocular skin basal cell carcinoma cases treated with ECT.

	Age (year)/gender	Periorbital localization	Other localization	Size of periocular tumor/s (mm)	Primary/recurrent/previous treatments	Route of Bleomycin administration	Type of electrode/average current(A)	Follow-up (month)	Results
1	44/M	upper eyelid		35 × 12 × 3	primary	it	row needle/3–5	16	CR
2	64 M	lower eyelid,	fronto-temporal region and cheek	43 × 27 × 1	recurrent/multiple surgeries, vismodegib	iv	hexagonal/4–6 row needle/2–5	36	CR
3	72/M	lower eyelid		10 × 10 × 1	recurrent/surgery	it	row needle/1.5–3	18	CR
4	71/M	medial canthal region		7 × 9 × 2	recurrent/surgery	it	row needle/7–10	16	CR
5	77/F	lower eyelid,	both hands, nose	13 × 10	recurrent after surgery	iv	hexagonal/4–6	21	CR
6	35/F	eyebrow		25 × 10	recurrent/surgery	iv	hexagonal/2–3	56	CR
7	11/F	upper eyelid, lower eyelid, both canthal regions	nose, perioral skin, frontotemporal area	6 × 8 ; 12 × 5	recurrent/surgery	iv	row needle/2–3	20	CR
8	80/F	both lower eyelids, medial canthi	head-neck, back	15 × 108 × 5	recurrent/surgery	iv	row needle/4–6	20	CR
9	62/F	lateral canthus, upper eyelid	head-neck, back	8 × 6 5 × 4 × 2	recurrent/surgery	iv	hexagonal/3–6	55	CR
10	72/M	lower eyelid, medial canthal region	head-neck, chest, upper extremities	7 × 5 × 3; 2 × 3 × 1	recurrent/surgery	iv	row needle/1,5–3	15	CR
11	86/M	lateral canthal region	trunk, head-neck, extremities	25 × 25	primary	iv	row needle/2–3	16	CR
12	65/M	medial canthal region	trunk, head-neck, extremities	14 × 14 × 2	primary	iv	row needle/1.5–3	15	CR

Basal cell carcinomas in other localizations were also treated with ECT. (CR: complete response; it: intratumoral; iv: intravenous).

The route of bleomycin administration was intratumoral in 3 patients and intravenous in 9 patients. The intratumoral dose of bleomycin was calculated according to the size of the lesion (250–1000 IU/cm^3^), the systemic dose was 15000 IU/m^2^. Safety margins around the tumors were treated in all cases. Intravenous administration was indicated in patients with multiple tumors including those with extra-periocular skin localization.

The following local adverse events were noticed in the early postoperative period: spontaneously resolving hyperemia and mild edema could be observed in 80% of the patients 3–4 days after the treatment, while mild pain was developed in 50% of the patients.

**Figure 1:**
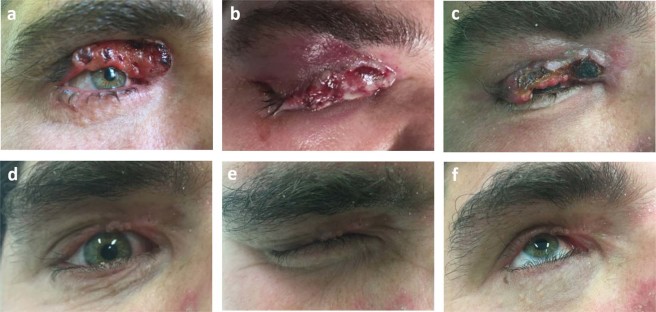
Electrochemotherapy (ECT) in the treatment of primary upper eyelid basal cell carcinoma (patient No 1). (**a**) Clinical appearance before the treatment. (**b**) Swelling of the treated area two days after ECT. (**c**) Partial response 16 days after ECT. (**d**) Complete response 2 months after one session of ECT. (**e**,**f**) Preserved function of the upper eyelid.

**Figure 2:**
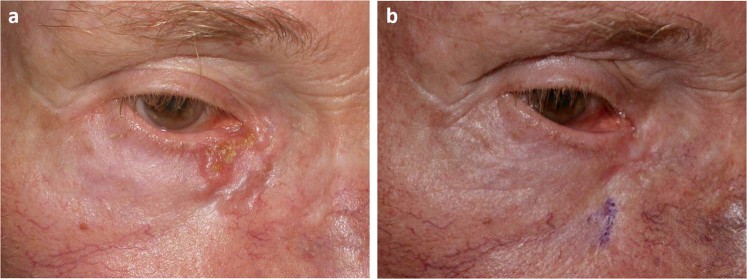
Basal cell carcinoma in the medial canthal region treated with 1 session of ECT (patient No 12). (**a**) Clinical appearance before the treatment. (**b**) Complete response 6 months after the ECT treatment.

**Figure 3:**
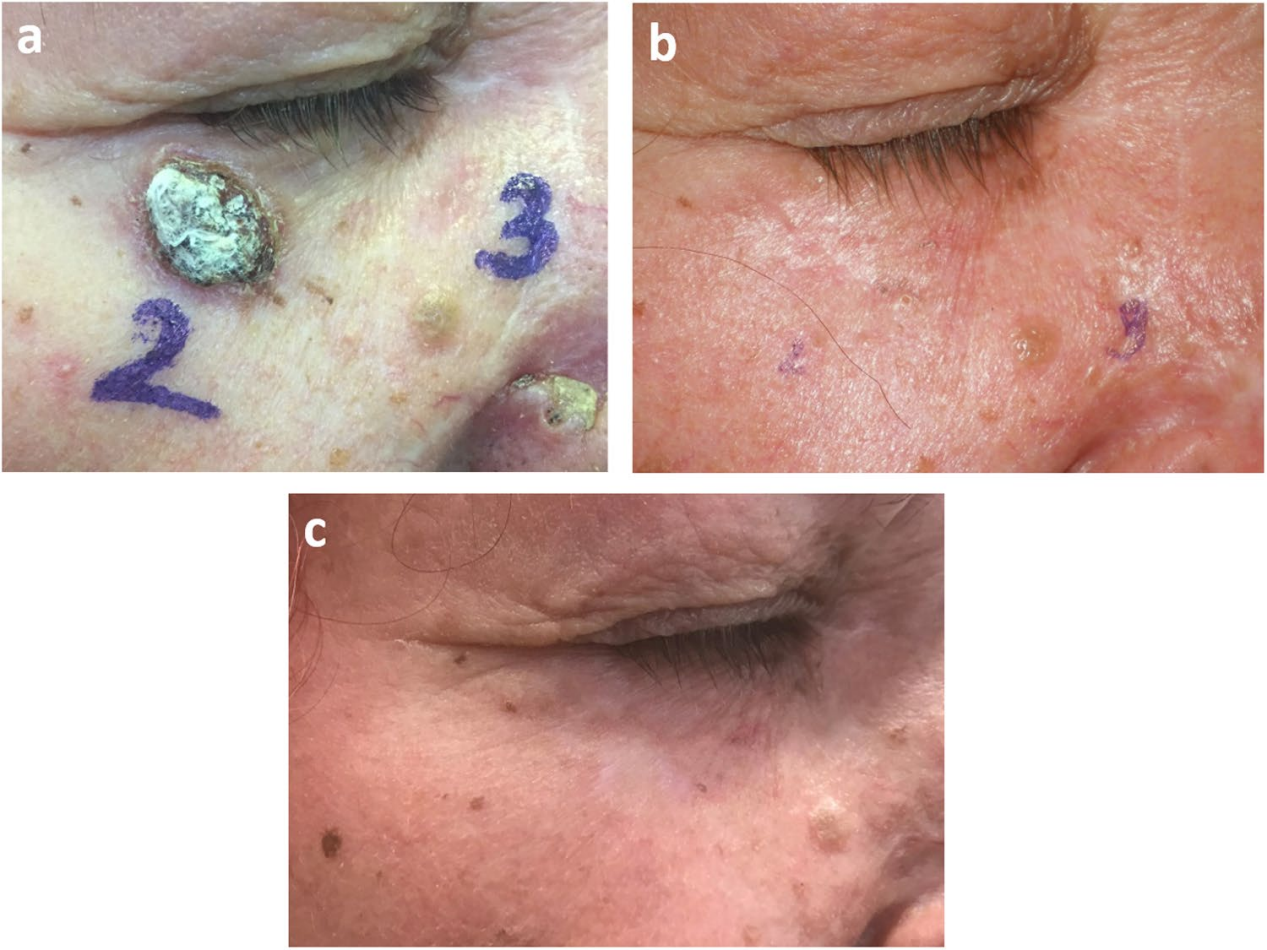
Recurrent basal cell carcinomas on the right lower eyelid and nasal area (patient No 5). (**a**) Clinical appearance before ECT treatment. (**b**) Complete response was achieved with 1 session of ECT. Clinical appearance 12 months after the treatment. (**c**) The treated skin region is tumor free 16 months after the ECT treatment.

**Figure 4:**
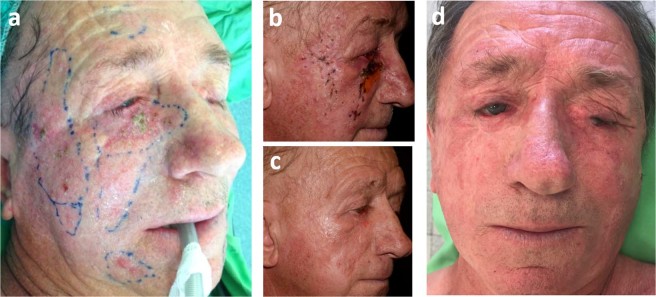
Recurrent multiple basal cell carcinomas involving the right lower eyelid, cheek and fronto-temporal skin areas (patient No 2). (**a**) Treatment plan (with safety zones) (**b**) Marks of the needle electrodes and crusts on the right temple, cheek and lower eyelid 10 days after ECT (**c**) Complete response 3 months after two sessions of ECT (**d**) Complete response 24 months after the ECT and the correction of ectropion of the right lower eyelid.

**Figure 5:**
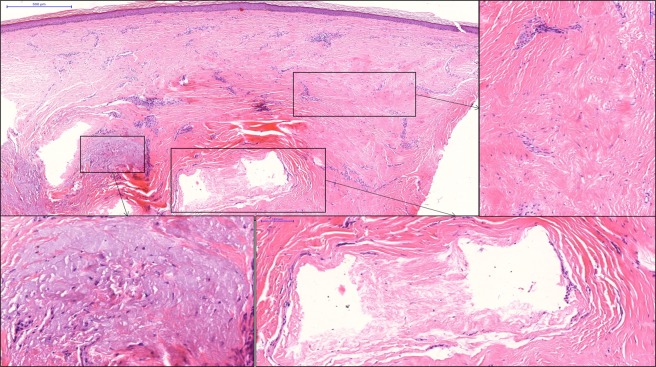
Histological findings in the residual scar. Low power micrograph of treated skin (H&E; OM112x, scanning slide). The entire dermis is affected by diffuse fibrosis (insert upright) with residual basophilic solar elastin degeneration (insert down left). The optically empty pseudocystic spaces seen in the surrounding area can be the result of tumor destruction caused by ECT treatment (insert down right).

Nine patients (75%) with recurrent tumors and 3 patients (25%) with primary tumors were treated. Eight patients (66.6%) had tumors not only in the periocular area, but also in other extraorbital skin locations. CR was achieved in all 12 cases (100%) in the periocular localization. These responses were reached with 1 session of ECT in 7 cases (58.3%), with 2 treatment sessions in 3 cases (25%), while 4 sessions were required in 1 patient (8.3%) and 5 sessions in 1 case (8.3%). Figure 1 demonstrates the treatment results of a locally advanced primary BCC on the upper eyelid (patient No 1) while Fig. 2 shows the results of ECT treatment of a medial canthal BCC (patient No 12). In these cases, ECT was the first choice of treatment and CR could be achieved with 1 treatment session. Figure 3 shows the ECT treatment results of patient No. 5 with multiple recurrent tumors on the right lower eyelid and nose. CR could be achieved not only in the periocular and nasal localizations but also on the hands (this later results are not shown). Higher number of treatment sessions were associated with genetic susceptibility to BCCs: one patient with Gorlin-Goltz syndrome required 4 sessions of ECT while 1 patient with xeroderma pigmentosum was treated with 5 applications of ECT. Ectropion caused by post-ECT scar was developed in 3 patients (cases 2, 3 and 7; 25%), which had to be corrected surgically. In case of patient No. 2, besides continuous progression of the tumor during vismodegib treatment, intolerable adverse events hindered long-term administration of the drug. After 2 sessions of ECT treatment, CR of the tumors on the eyelid and in other facial locations could be achieved (Fig. 4). Biopsies were taken from the treated areas 6–12 months after the ECT treatment (except for 1 patient who refused sampling). Histopathological examination showed no tumor remnant in the samples (Fig. 5).

## Discussion

Increasing incidence of UV radiation-induced malignancies can be observed during the past two decades. Since periocular skin is one of the most sun-exposed surface of the human body, increasing number of BCCs in this anatomical region causes emerging health issues^20,21^. Various factors may influence the success of tumor treatment. Besides the size and number, location and histological subtype of the tumor, the patient’s age, co-morbidities, and general condition can influence the outcome and tolerability of the treatment. Adequate management of locally advanced or recurrent periocular BCCs are particularly difficult, as not only oncological, but also functional and cosmetic aspects should be considered when the most appropriate treatment modalities are planned. The periocular skin-eyelid area has a distinguished role in the protection of the ocular surface and thus in the maintenance of clear vision. Unfavorable outcome of the treatment can result in loss of vision and distorting scars can deeply impact the patient’s quality of life^22,23^.

There is increasing evidence about the effectiveness of ECT in various types of skin tumors including those, located in the head and neck region^15,24^. New guideline has been published to delineate the clinical and technical parameters of the treatment^25^. In general, ECT has many advantages in the treatment of BCC. In case of elderly patients with various co-morbidities, this treatment represents an option with low systemic burden and fast recovery. ECT can be used in previously irradiated skin areas, where the excision of the recurrent tumor is difficult and the success of other interventions are also limited. Healthy tissues surrounding the tumor can be saved due to the cell-type selectivity of the treatment, which is particularly important in the periocular region, where the extension of surgical interventions is limited. As the effect of ECT on normal tissue around the tumor is minimal, it enables the treatment of safety margins which has great importance especially in BCCs with ill-defined borders and in recurrent cases. Further advantages of the treatment are the repeatability and the low rate of systemic adverse events. Patients with non-melanoma skin cancers often present with numerous tumors. During ECT multiple skin tumors can be treated in the same session.

In contrast to the continuously gathering information about the effectiveness of ECT in other skin areas, only few patients with periocular BCC treated with ECT have been reported so far. According to our literature search, Landström and colleges reported two patients with medial canthal BCC and Salwa and colleges reported three patients with primary periocular BCC successfully treated with ECT using intratumoral bleomycin^13,17^. In the European Research on Electrochemotherapy in the Head and Neck Cancer (EURECA) project published in 2016, 5 tumor nodules involving the eyelid-orbit region were included without detailed description of the lesions^26^. Both the tumor types and the response rates of head and neck malignancies were pooled, thus no further consequences could be gained regarding the success of the treatment in the periocular region.

In the present study, we report the results of ECT treatments in patients with challenging eyelid-periocular BCC conditions (Table 1). CR could be achieved with ECT treatment in locally advanced primary eyelid BCCs and in recurrent cases. Patients with Gorlin-Goltz syndrome and xeroderma pigmentosum could also be effectively treated with this modality. Furthermore, ECT could be used successfully in a vismodegib-resistant case. Mild to moderate scarring could be observed in the treated skin areas. Lower eyelid ectropion caused by contracting scars was developed in 3 patients, which could be successfully corrected with surgery. Overall, ECT could be used effectively in the treatment of BCC in the periocular region. Excellent tumor control could be achieved with good functional and cosmetic results with short interval follow-up. We did not observe systemic adverse events of the treatment. This study has several limitations. With longer follow-up and with higher number of treated patients, the tumor recurrence rate and the frequency of the complications may be altered. High risk BCC patients were selected to this study, which condition is associated with higher tumor recurrence rate. The follow-up time in our study (median: 19 months) is not long enough for the assessment of the recurrence-related efficacy of the treatment, therefore only moderate consequences can be deduced so far. Further studies are urgently needed in order to establish the role of ECT in the treatment of periocular skin-eyelid BCCs and to delineate the subpopulation of patients for whom ECT can be most beneficial.

## Data Availability

All data generated or analysed during this study are included in this article.
